# Predictors of functional outcome after endovascular thrombectomy in patients with large ischemic core based on DWI-ASPECTS

**DOI:** 10.1186/s12883-026-04967-6

**Published:** 2026-05-18

**Authors:** Juan He, Kaixuan Ren, Yanchi Xu, Boxuan Zhao, Liyang Yue, Yingge Wang, Tieyu Tang, Xiaoliang Xie, Wei Wang, Yi Zhao, Zhensheng Liu

**Affiliations:** 1 Department of Neurology, Affiliated Hospital of Yangzhou University, Yangzhou University, Yangzhou, Jiangsu 225100 China; 2 Department of Medical Imaging, Affiliated Hospital of Yangzhou University, Yangzhou University, Yangzhou, Jiangsu 225100 China; 3 Department of Neurology, The First People’s Hospital of Suqian, Jiangsu, 226001 China; 4 Department of Interventional Radiology, Affiliated Hospital of Yangzhou University, Yangzhou University, Yangzhou, Jiangsu 225100 China

**Keywords:** DWI-ASPECTS, Large ischemic core, ACALVOS, Endovascular thrombectomy, SICH

## Abstract

**Background:**

Several clinical trials have shown the benefit of endovascular thrombectomy (EVT) in patients with large ischemic core infarction. However, the imaging selection modalities used for patient selection have differed across studies. This study aimed to assess the efficacy, safety, and prognostic factors of EVT in patients with large ischemic core selected only on the basis of Diffusion-Weighted Imaging Alberta Stroke Program Early CT Score (DWI-ASPECTS).

**Method:**

This single-center study, conducted from 2019 to 2024, included patients with anterior circulation acute large vessel occlusion and stratified them into three groups according to DWI-ASPECTS: non-large ischemic core (≥ 6) treated with EVT (*n* = 77), large ischemic core (3–5) treated with EVT (*n* = 91), and large ischemic core (3–5) treated with medical management alone (*n* = 70). The primary outcome was functional independence at 90 days, defined as a modified Rankin Scale (mRS) score of 0–2. Secondary endpoints included symptomatic intracranial hemorrhage (sICH) within 48 h and mortality within 90 days. Multivariate binary logistic regression was performed to identify factors associated with functional independence in the large-ischemic-core EVT group.

**Results:**

Patients with large ischemic core treated with EVT had a significantly higher rate of 90-day functional independence than those who received medical management (53.8% vs 28.6%, *P* = 0.001). No significant differences in sICH or mortality were observed between the large-ischemic-core EVT and medical management groups. However, compared with patients with non-large ischemic core treated with EVT, those with large ischemic core treated with EVT had a lower rate of functional independence (53.8% vs 70.1%, *P* = 0.039). In the large ischemic core EVT group, intravenous thrombolysis (OR 0.164, *P* = 0.018) and parenchymal hematoma type 2 (PH2) hemorrhage (OR 25.641, *P* = 0.012) were independent predictors of 90-day outcomes.

**Conclusion:**

In this cohort, EVT was associated with improved 90-day functional outcomes in patients with large ischemic core (DWI-ASPECTS 3–5) compared with medical management alone, without a statistically significant increase in sICH or mortality. Intravenous thrombolysis and PH2 hemorrhage were identified as independent predictors of outcome. These results require further confirmation in larger and adequately powered studies.

## Introduction

Endovascular thrombectomy (EVT) is a recommended treatment for large-vessel occlusion (LVO) in the anterior circulation [1–4]. According to the 2026 American Heart Association/American Stroke Association (AHA/ASA) guideline for the early management of acute ischemic stroke [5], patients with anterior circulation LVO, Alberta Stroke Program Early Computed Tomographic Score (ASPECTS) 3–5, and symptom onset within 6 h are now strongly recommended to receive EVT (Class of Recommendation 1, Level of Evidence A). For patients presenting within 6–24 h of onset with ASPECTS 3–5, EVT is also recommended (COR 1, LOE A). These updated recommendations differ from earlier guidelines [6], which limited EVT eligibility to patients with ASPECTS ≥ 6. The revised guidelines are supported by multiple randomized controlled trials (RCTs) [7–11], all of which reported improved functional outcomes with EVT in patients with a large ischemic core compared with medical management alone.

Despite these advances, the optimal imaging selection modality for identifying a large ischemic core remains debated. Non‑Contrast Computed Tomography (NCCT)‑based ASPECTS is widely available but has limited inter‑rater reliability and sensitivity for early detecting ischemic change [12]. Computed Tomography Perfusion (CTP)-based ASPECTS is frequently used in clinical practice. However, CTP requires contrast administration, involves additional radiation exposure, and may overestimate infarct core size during the early phase of stroke [13, 14]. In comparison, diffusion-weighted imaging (DWI)-ASPECTS effectively identifies the location and extent of early-stage lesions and shows a strong correlation with the final infarct volume [15]. DWI-ASPECTS has been widely used to assess hemorrhagic risk after thrombolysis, evaluate EVT response, and predict stroke prognosis in patients [16–18]. Therefore, preoperative DWI‑ASPECTS may serve as a suitable imaging approach for patients likely to benefit from EVT.

In this retrospective study, patients with acute anterior circulation large artery occlusive stroke (ACALVOS) treated at our institution within 24 h of symptom onset were evaluated. Patients were categorized into three groups based on baseline DWI‑ASPECTS and whether EVT was performed: non‑large core (≥ 6) treated with EVT, large ischemic core (3–5) treated with EVT, and large ischemic core (3–5) receiving medical management alone. The primary objective was to assess the efficacy and safety of EVT in patients with large ischemic core selected only by DWI‑ASPECTS, and to identify independent predictors of 90‑day functional outcome. This study sought a preliminary analysis of the regulatory factors associated with EVT prognosis in this specified patient population.

## Methods

### Sample population

**Fig. 1:**
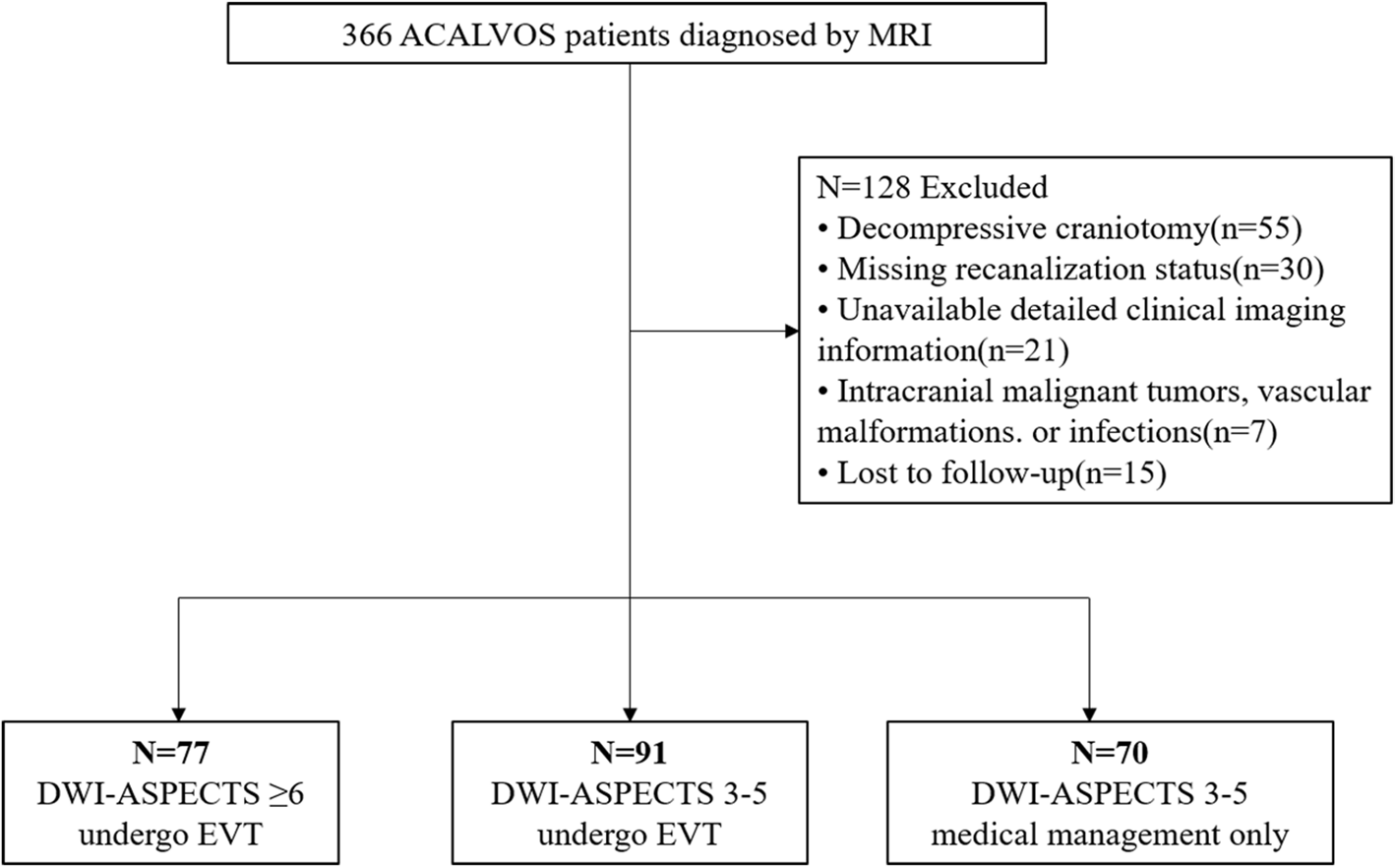
Study flow chart. ACALVOS, Anterior Circulation Acute Large Vessel Occlusion Stroke; DWI-ASPECTS, Diffusion-Weighed Imaging Alberta Stroke Program Early CT Score

Consecutive patients admitted to our hospital between July 2019 and July 2024 were retrospectively screened for eligibility. Patients with an unknown time of symptom onset were eligible if they presented within 24 h of being last known well and demonstrated a diffusion-weighted imaging (DWI) lesion without a corresponding lesion on fluid-attenuated inversion recovery (FLAIR) imaging, indicating that less than 4.5 h had likely elapsed since stroke onset [19, 20]. Participants were included if they met the following criteria: (1) age ≥ 18 years; (2) National Institutes of Health Stroke Scale (NIHSS) score [21] ≥ 4 at admission; (3) pre-stroke modified Rankin Scale (mRS) score [22] ≤ 1; (4) evidence of successful blood flow recanalization during intraoperative angiography, defined as a modified Thrombolysis in Cerebral Infarction (mTICI) grade [23, 24] of 2b or 3; and (5) availability of complete clinical and imaging data. Exclusion criteria were as follows: (1) pre-stroke mRS score > 1; (2) presence of midline shift, cerebral herniation, mass effect, or high hemorrhagic risk; (3) coexisting intracranial conditions, including malignant tumors, vascular malformations, or infections; (4) severe comorbid life-threatening or disabling conditions (e.g., advanced malignancy, end-stage renal or hepatic failure) that could affect survival or functional outcome assessment; (5) refusal of EVT by patients or their families; and (6) poor MRI quality due to motion artifacts in patients who were non-compliant or uncooperative during MRI examination, that prevented reliable DWI-ASPECTS assessment. Moreover, patients with DWI-ASPECTS scores of 3–5 who did not undergo EVT during the same period were included as a medical management control group, provided they met the same inclusion and exclusion criteria except for EVT treatment. Reasons for not undergoing EVT included contraindications, delayed hospital presentation beyond the treatment window, or refusal by the patient or family. A detailed flowchart of patient selection is presented in Fig. 1.

### Clinical information

The following clinical variables were collected: age, sex, vascular risk factors (hypertension, hyperlipidemia, diabetes mellitus, atrial fibrillation, smoking history, and previous stroke), baseline systolic and diastolic blood pressure, site of vascular occlusion (internal carotid artery, M1 segment of the middle cerebral artery, or M2 segment of the middle cerebral artery), mTICI grade, use of intravenous thrombolysis, stent implantation, presence of reocclusion or tandem occlusion, admission blood glucose level, onset-to-reperfusion time (ORT), and baseline NIHSS score.

### Imaging information

**Fig. 2:**
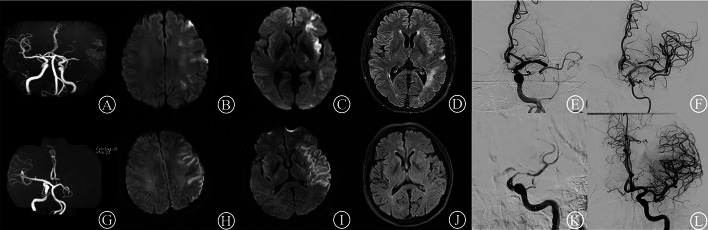
Representative images of different patients with ACALVOS. **A**-**F**: A patient with M1 segment occlusion of the left middle cerebral artery and DWI-FLAIR mismatch. The preoperative DWI-ASPECTS was 3 points, and the post-operative mTICI grade was 2b following EVT; Figure **G**-**L**: A patient with left internal carotid artery intracranial segment occlusion and DWI-FLAIR mismatch. The preoperative DWI-ASPECTS was 4 points, and the post-operative mTICI grade was 3 following EVT

All medical image captures were performed using a Siemens Verio 3.0 T MR imaging system (Siemens, Erlangen, Germany). The scan lasted 4 min and 50 s and included DWI, magnetic resonance time-of-flight angiography (TOF-MRA), and T2-FLAIR. This study assigned a weight of 1 to each of the 10 locations evaluated across two successive layers within the perfusion territory of the middle cerebral artery on DWI. A point was subtracted for each region that showed involvement. The overall score ranged between 0 and 10, with 0 indicating infarction in all regions and 10 suggesting the absence of infarction across all 10 regions [25]. Baseline infarct volume was quantified using ITK-SNAP software (version 3.8). Infarct regions were manually segmented on DWI images, and infarct volume (mL) was automatically calculated from the segmented regions. The DWI-FLAIR mismatch was defined as the presence of a DWI lesion without a corresponding FLAIR hyperintensity or with significantly smaller FLAIR changes relative to DWI [20]. A representative case is shown in Fig. 2.

A post-operative CT scan was also performed in this investigation using a Siemens third-generation dual-source Force CT scanner (Germany). The virtual non-contrast (VNC) scan showed a relatively high-density area, suggesting subsequent bleeding following surgery. Two experienced neuroradiologists, blinded to clinical data and patient outcomes, independently reviewed the DWI images and assigned DWI-ASPECTS scores. Infarct segmentation was also performed independently. Any discrepancies were settled after consulting with two experts.

### Measurements and outcomes

EVT was performed according to standard clinical protocols. In eligible patients presenting within 4.5 h of symptom onset, EVT was administered in combination with intravenous recombinant tissue-type plasminogen activator (rt-PA) thrombolysis. For patients with contraindications to intravenous thrombolysis or those considered unlikely to achieve recanalization after thrombolytic therapy, neurointerventional physicians assessed neurological deficits and, after discussion with the patient's family, determined whether to proceed directly with EVT.

Using the European Cooperative Acute Stroke Study (ECASS) classification system [26], sICH was defined as the presence of any intracranial hemorrhage with a neurological worsening of 4 or more points on the NIHSS, we next stratified the sICH into 4 discreet categories, namely, hemorrhagic infarction type 1(HI1), hemorrhagic infarction type 2 (HI2), parenchymal hematoma type 1 (PH1) and parenchymal hematoma type 2 (PH2). Efficacy was evaluated using the mRS score and the rate of functional independence at 90 days, obtained through telephone follow-up or outpatient assessment [22]. Participants who were lost to follow-up were eliminated. A score of 0 to 2 indicated functional independence, whereas a score of 3 to 6 indicated an adverse prognosis. The safety endpoint was assessed by sICH within 48 h, and all-cause mortality was evaluated at 90 days [27].

### Statistical analysis

All statistical analyses were performed using SPSS version 21.0 (IBM Corp., Armonk, NY, USA). Categorical variables are presented as counts and percentages, while continuous variables are expressed as mean ± standard deviation or median (interquartile range [IRQ]), as appropriate. Inter-rater reliability for infarct volume measurements was assessed using the intraclass correlation coefficient (ICC). The relationship between DWI-ASPECTS score and infarct volume was evaluated using Spearman correlation analysis. In the univariate analysis, the Chi-square test, Fisher's exact test, Student's *t*-test, and Mann–Whitney *U* test were used as appropriate to assess differences in baseline profiles and prognostic markers between the three cohorts. The mRS score was expressed as median (IQR) and compared using the Mann–Whitney *U* test. Using the 90-day mRS score, large ischemic core patients were classified into a functional independence (0–2 points) and an unfavorable-prognosis (3–6 points) cohort. This study employed univariate analysis to assess changes in clinical and imaging characteristics across groups before and during EVT. Variables that reached significance (*P* < 0.05) in univariate analysis were included in a multivariate binary logistic regression analysis to identify independent risk factors for poor prognosis in patients with large ischemic cores. The propensity score covariate adjustment method was employed to alleviate confounding variables. Pre‑stroke mRS was not included as a covariate because all patients met the inclusion criterion of mRS ≤ 1, resulting in minimal variability. A *P* value < 0.05 denotes statistical significance.

## Results

### Comparison of baseline clinical demographics and prognostic markers between the three cohorts

A total of 238 patients were included in the study, comprising 77 patients with DWI-ASPECTS ≥ 6 treated with EVT (Group A), 91 patients with DWI-ASPECTS 3–5 treated with EVT (Group B), and 70 patients with DWI-ASPECTS 3–5 treated with medical management alone (Group C). Baseline characteristics are presented in Table 1. Among patients who underwent EVT, 135 presented within 6 h of symptom onset, whereas 33 presented between 6 and 24 h. All baseline variables were assessed by univariate analysis. No significant differences were observed between Group A and Group B with respect to age, sex, time of onset > 6 h, baseline blood pressure, history of hypertension, diabetes, smoking, atrial fibrillation, previous stroke, baseline NIHSS score, or admission blood glucose level. Similarly, compared with Group B, patients in Group C showed no significant differences in age, sex, vascular risk factors, baseline NIHSS score, infarct volume, and DWI-ASPECTS distribution (all *P* > 0.05) (Table 1). Spearman correlation analysis demonstrated a moderate inverse correlation between DWI-ASPECTS and infarct volume (*r*ₛ = − 0.502, *P* < 0.001), indicating that lower ASPECTS scores were associated with larger infarct volumes.

**Table 1:** Univariate analysis of baseline characteristics for the three patient groups

Variable	No. (%)			t/z/χ^2^ */#	*P* value */#
	ASPECTS ≥ 6 (*n* = 77)	ASPECTS 3 ~ 5 with EVT (*n* = 91)	ASPECTS 3 ~ 5 non-EVT (*n* = 70)		
Age, mean (SD), y	67.1 ± 11.5	66.5 ± 12.5	67.7 ± 13.4	0.321/0.556	0.484/0.579
Male	48 (62.3)	67 (73.6)	48 (68.6)	0.246/0.495	0.135/0.488
Time of onset > 6 h	12 (15.6)	21 (23.1)	22 (31.4)	1.483/1.410	0.247/0.282
Blood pressure, mean (SD), mmHg					
Systolic	153.4 ± 27.2	151.5 ± 26.4	148.6 ± 23.7	0.461/0.727	0.571/0.468
Diastolic	87.9 ± 13.7	86.7 ± 14.2	87.6 ± 13.3	0.542/0.399	0.525/0.691
Hypertension	55 (71.4)	59 (64.8)	44 (62.9)	0.831/0.067	0.409/0.869
Diabetes	24 (31.2)	22 (24.2)	12 (17.1)	1.026/1.175	0.386/0.332
Current smoking	20 (26.0)	22 (24.2)	18 (25.7)	0.072/0.050	0.859/0.855
Atrial fibrillation	30 (39.0)	37 (40.7)	22 (31.4)	0.050/1.452	0.875/0.251
Previous stroke	12 (15.6)	21 (23.1)	9 (12.9)	1.483/0.133	0.247/0.807
Baseline NIHSS score, median (IQR)	12 (8, 18)	15 (10, 20)	12 (7, 18)	1.588/1.194	0.112/0.233
FBG, mean (SD), mmol/L	8.3 ± 3.8	7.7 ± 3.5	7.5 ± 2.9	1.045/0.441	0.677/0.660
DWI-ASPECTS, median (IQR)	7 (6, 8)	3 (4, 5)	4 (4, 5)	-/0.747	-/0.455
Infarct volume, mean (SD), ml	36.1 ± 15.6	88.6 ± 63.8	75.77 ± 25.8	-/1.746	-/0.083

*Abbreviations*: *NIHSS* National Institutes of Health Stroke Scale, *FBG* Fasting blood glucose

*, Group A vs Group B

#, Group B vs Group C

### Evaluation of surgical information and prognostic markers between the three cohorts

#### Comparison between EVT-treated patients with large and non-large ischemic cores (Group B vs. Group A)

**Fig. 3:**
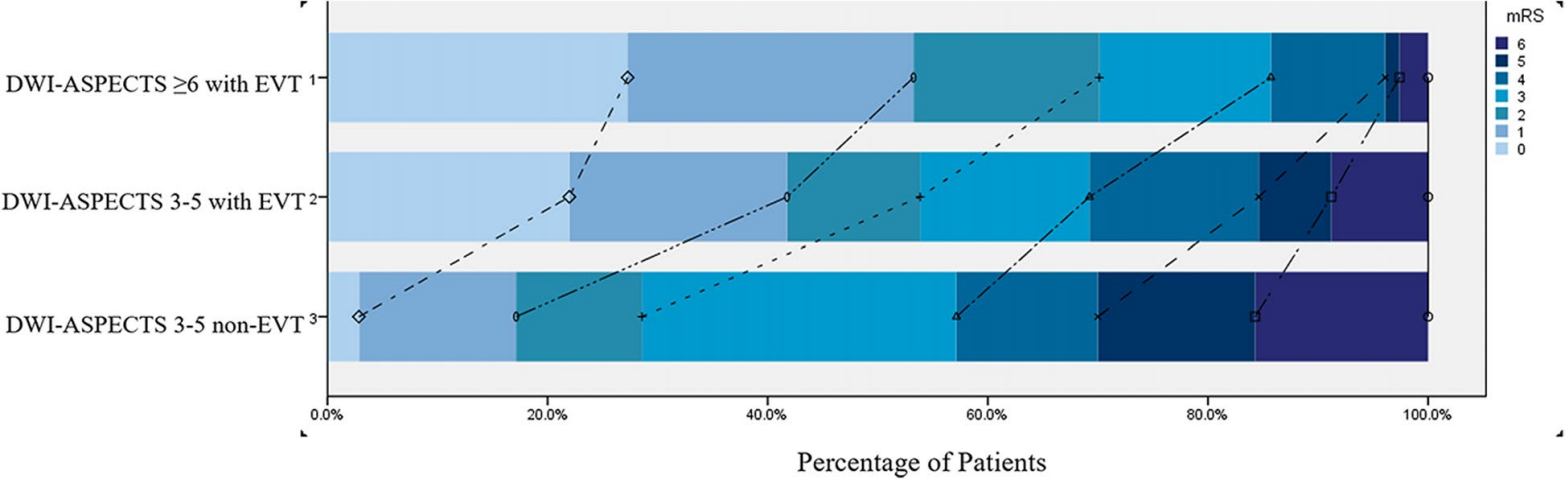
Distribution of modified Rankin Scale (mRS) score at 90 days after discharge. DWI-ASPECTS, Diffusion-Weighed Imaging Alberta Stroke Program Early CT Score; mRS, modified Rankin Scale

Tandem occlusions were more frequent in patients with a large ischemic core than those without (24.2% versus 11.7%, *P* = 0.046). The 90-day mRS score demonstrated a significant difference between the two groups. The median mRS score was 2 in the large-ischemic-core group and 1 in the non-large-ischemic-core group. Moreover, the proportion of patients achieving functional independence at 90 days (mRS 0–2) was lower in the large ischemic core group than in the non-large ischemic core group (53.8% vs. 70.1%, *P* = 0.039) (Fig. 3). No significant differences were observed in 90-day intravenous thrombolytic bridging therapy, ORT, occlusion site, sICH, or all-cause mortality between the two groups (Table 2). The subgroup analysis revealed no differences in EVT efficacy and safety parameters between patients with large ischemic cores and those with non-large ischemic cores at time of onset > 6 h (Table 3).

**Table 2:** Univariate analysis of surgical data and prognostic indicators for the three patient groups

Variable/Outcome	No. (%)			t/z/χ^2^ */#	*P* value */#
	ASPECTS ≥ 6 (Group A, *n* = 77)	ASPECTS 3 ~ 5 with EVT (Group B, *n* = 91)	ASPECTS 3 ~ 5 non-EVT (Group C, *n* = 70)		
rt-PA Intravenous thrombolysis	28 (36.4)	22.0 (24.2)	13 (18.6)	2.964/0.730	0.093/0.444
ORT (min, median (IQR)	373 (296, 510)	443 (310, 600)	-	−1.260/-	0.205/-
Occlusion site					
ICA	28 (36.4)	41 (45.1)	21 (30.0)	1.302/3.787	0.274/0.072
M1	40 (51.9)	44 (48.4)	41 (58.6)	1.986/3.457	0.186/0.067
M2	9 (11.7)	6 (6.6)	8 (11.4)	1.331/1.165	0.286/0.398
Tandem occlusion	9 (11.7)	22 (24.2)	-	4.322/-	0.046/-
Stent-retriever first-line	17 (22.1)	16 (17.6)	-	0.534/-	0.560/-
sICH					
HI1	7 (9.1)	8 (8.8)	3 (4.3)	0.005/1.262	1.000/0.351
HI2	4 (5.2)	10 (11.0)	3 (4.3)	1.833/2.395	0.263/0.151
PH1	4 (5.2)	4 (4.4)	3 (4.3)	0.059/0.001	1.000/1.000
PH2	5 (6.5)	8 (8.8)	5 (7.1)	0.308/0.145	0.464/0.777
mRS score at 90 d, median (IQR)	1 (0, 3)	2 (0, 4)	3 (2, 5)	−3.108/3.665	0.002/< 0.001
mRS score of 0–2 at 90 d	54 (70.1)	49 (53.8)	20 (28.6)	4.662/10.321	0.039/0.001
Mortality within 90 days	2 (2.6)	8 (8.8)	12 (17.4)	2.858/2.537	0.111/0.148

*Abbreviations*: *rt-PA* recombinant tissue plasminogen activator, *ORT* Onset-to-reperfusion time, *ASPECTS* Alberta Stroke Program Early Compute Tomography Score, *ICA* internal carotid, *sICH* symptomatic intracranial hemorrhage, *HI* Hemorrhagic infarction, *PH* parenchymal hemorrhage, *mRS* modified Rankin Scale

*, Group A vs Group B

#, Group B vs Group C

**Table 3:** Prognostic variations in endovascular thrombectomy for two patient groups with onset time exceeding 6 h

Variable/Outcome	Time of onset > 6 h		z/χ^2^	*P* value
	ASPECTS ≥ 6 (*n* = 12)	ASPECTS 3 ~ 5 (*n* = 21)		
mRS score at 90 days	4 (3, 4)	4 (4, 5)	−1.154	0.248
mRS score of 0–2 at 90 days	10 (83.3)	13 (61.9)	1.660	0.259
sICH within 48 h	3 (25.0)	6 (28.6)	0.049	1.000
Mortality within 90 days	1 (8.3)	1 (4.8)	0.171	1.000

*Abbreviations*: *ASPECTS* Alberta Stroke Program Early Compute Tomography Score, *mRS* modified Rankin Scale, *sICH* Symptomatic intracranial hemorrhage

#### Comparison between large ischemic core patients with and without EVT (Group B vs. Group C)

Among patients with large ischemic core infarction, those treated with EVT showed significantly better functional outcomes at 90 days compared with those receiving medical management alone. The median mRS score was 2 (IQR 0–4) in the EVT group versus 3 (IQR 2–5) in the medical management group (*P* < 0.001). The proportion of patients achieving functional independence (mRS 0–2) was also higher in the EVT group (53.8% vs. 28.6%, *P* = 0.001) (Fig. 3). The rates of sICH were numerically higher in the EVT group but did not reach statistical significance (HI2: 11.0% vs. 4.3%, *P* = 0.151; PH2: 8.8% vs. 7.1%, *P* = 0.777). Mortality within 90 days was 8.8% in Group B compared with 17.4% in Group C (*P* = 0.148) (Table 2). These findings suggest that EVT may improve functional outcomes in patients with large ischemic core infarction without a statistically significant increase in serious hemorrhagic complications or mortality.

However, the comparison of mortality between groups was limited by the relatively small number of events and insufficient statistical power. Although no statistically significant difference in sICH was observed, the numerically higher rate of hemorrhagic transformation in the large-ischemic-core EVT group, together with the limited sample size, warrants cautious interpretation.

### Analysis of prognosis-regulating factors among the large ischemic core cohort

Among the patients with large ischemic core infarction who underwent EVT (Group B), 49 (53.8%) had a favorable prognosis at 90 days, while 42 (46.2%) had a poor prognosis. Univariate analysis showed significant differences between the two groups in baseline NIHSS scores, intravenous thrombolytic bridging therapy, and the occurrence of HI2 and PH2 intracranial hemorrhages (Table 4). Subsequent multivariate analysis showed that the intravenous thrombolytic bridging therapy (OR = 0.164, 95% CI: 0.037–0.733; *P* = 0.018) and PH2 type sICH (OR = 25.641, 95% CI: 2.024–324.889; *P* = 0.012) were independent factors associated with 90-day prognosis in patients with large ischemic core infarction treated with EVT (Table 5). The OR < 1 for intravenous thrombolysis indicates a protective effect, corresponding to lower odds of a poor functional outcome. The estimates for PH2 and HI2 should be interpreted cautiously due to the small number of events and potential model overfitting.

**Table 4:** Univariate analysis of prognostic factors in the large ischemic core group

Variable	No. (%)		t/z/χ^2^	*P* value
	mRS0-2 (*n* = 49)	mRS > 2(*n* = 42)		
Age, mean (SD), y	64.3 ± 13.7	69.0 ± 10.7	−1.838	0.691
Male	38 (77.6)	29 (69.0)	0.842	0.475
Hypertension	30 (61.2)	29 (69.0)	0.607	0.512
Diabetes	11 (22.4)	11 (26.2)	0.173	0.807
Current smoking	10 (20.4)	12 (28.6)	0.822	0.463
Atrial fibrillation	19.0 (38.8)	18.0 (42.9)	0.156	0.831
Previous stroke	3 (6.1)	7 (16.7)	2.571	0.178
FBG(mmol/)	7.3 ± 3.1	8.3 ± 3.8	−1.369	0.255
Baseline NIHSS, median (IQR)	14 (6, 18)	16.5 (12, 22)	−2.512	0.012
Time of onset > 6 h	13 (26.5)	8 (19.0)	0.713	0.460
DWI-ASPECTS			3.717	0.156
3	11 (22.4)	16 (39.1)		
4	14 (28.6)	13 (31.0)		
5	24 (49.0)	13 (31.0)		
rt-PA Intravenous thrombolysis	16 (32.7)	6 (14.3)	4.162	0.035
Tandem occlusion	11 (22.4)	11 (26.2)	0.173	0.807
Stent-retriever first-line	9 (18.4)	7 (16.7)	0.045	0.527
ORT(min, median (IQR)	410 (262, 570)	443 (310, 600)	−1.963	0.050
sICH				
HI1	2 (4.1)	6 (14.3)	2.937	0.137
HI2	2 (4.1)	8 (19.0)	5.179	0.040
PH1	2 (4.1)	2 (4.8)	0.025	1.000
PH2	1 (2.0)	7 (16.7)	6.033	0.022

*Abbreviations*: *rt-PA* Recombinant tissue plasminogen activator, *ORT* Onset-to-reperfusion time, *ASPECTS* Alberta Stroke Program Early Compute Tomography Score, *ICA* Internal carotid, *sICH* symptomatic intracranial hemorrhage, *HI* Hemorrhagic infarction, *PH* Parenchymal hemorrhage, *mRS* Modified Rankin Scale

**Table 5:** Multivariate analysis of prognostic factors in the large ischemic core group

	Unadjusted	*P* value	Adjusted*	*P* value
	OR (95% CI)		OR (95% CI)	
Baseline NIHSS	1.083 (1.012, 1.160)	0.021	0.164 (0.037, 0.733)	0.072
rt-PA Intravenous	0.150 (0.034, 0.656)	0.012	0.164 (0.037, 0.733)	0.018
HI2	7.656 (1.317, 44.506)	0.023	5.862 (0.988, 47.656)	0.051
PH2	25.056 (2.224, 282.255)	0.009	25.641 (2.024, 324.889)	0.012

*Abbreviations*: *NIHSS* National Institutes of Health Stroke Scale, *rt-PA* Recombinant tissue plasminogen activator, *HI* Hemorrhagic infarction, *PH* Parenchymal hemorrhage

^*^Results were adjusted for age, sex, time of onset > 6 h, DWI-ASPECTS, and Onset-to-reperfusion time

## Discussion

Several studies indicate that EVT benefits some patients with larger ischemic core strokes as determined by diagnostic imaging [7–11]. However, the imaging selection modalities used for patient selection have differed across studies. We conducted this study to evaluate the efficacy, safety, and prognostic factors of EVT in patients with large ischemic core selected solely by DWI‑ASPECTS. This retrospective study analyzed patients with ACALVOS who underwent EVT at our hospital within 24 h of symptom onset. Based on baseline DWI-ASPECTS, patients were categorized into three groups: non-large ischemic core (≥ 6) treated with EVT, large ischemic core (3–5) treated with EVT, and large ischemic core (3–5) treated with medical management alone. Three main findings were observed. First, EVT was associated with a significantly higher 90-day functional independence rate than medical management alone in patients with large ischemic core infarction (53.8% vs. 28.6%, *P* = 0.001). Second, EVT was not associated with a statistically significant increase in sICH or mortality, although some hemorrhagic subtypes showed higher point estimates. Third, intravenous thrombolysis, bridging therapy, and PH2-type sICH were identified as independent factors associated with 90-day outcome.

Regarding efficacy, our findings are consistent with recent randomized controlled trials [7–11]. The absolute benefit observed in this study (25.2%) appears comparable to, or even slightly higher than, that reported in these trials. Several factors may explain this difference. First, approximately 80% of the patients presented within 6 h of symptom onset, and some also received intravenous thrombolysis bridging therapy. Second, patients with midline shift, signs of herniation, mass effect, or high hemorrhagic risk, as determined by imaging or clinical evaluation, were excluded. Moreover, patients with DWI-ASPECTS 0–2 were not included in this study. Therefore, the efficacy and safety of EVT in this extreme subgroup remain unclear and require further investigation.

Regarding safety, no statistically significant increase in sICH or mortality was observed in patients treated with EVT, although the point estimates for certain hemorrhagic subtypes were higher. These findings differ from several previous studies that reported a higher risk of intracranial hemorrhage after thrombectomy in patients with large ischemic core infarction [10, 11, 28]. The lack of statistical significance in this study may be due to the limited sample size, low event rates, and insufficient statistical power. Therefore, these results should be interpreted with caution, and a possible increase in hemorrhagic risk associated with EVT in patients with a large ischemic core cannot be excluded. Moreover, subgroup analysis showed no significant differences in EVT efficacy or safety between patients with large and non-large ischemic cores with an onset duration of > 6 h. These findings suggest that EVT may remain both safe and effective in selected patients with a large ischemic core identified by DWI-ASPECTS, even in the extended time window.

The subgroup analysis of patients with large ischemic cores indicated that intravenous thrombolytic bridging treatment and PH2-type sICH were significant independent predictors of 90-day patient outcomes. Previous studies suggested that early intravenous thrombolysis should be considered before EVT in cases with large ischemic cores [29–32]. The sICH strongly contributes to the post-operative neurological decline and adverse patient prognosis [33]. The MR CLEAN (Multicenter Randomized Clinical Trial of Endovascular Treatment for Acute Ischemic Stroke in The Netherlands) trial [34] revealed that PH2-type sICH is closely associated with poor patient outcomes. Our findings are consistent with these results; intravenous thrombolysis bridging therapy was independently associated with favorable outcomes, whereas PH2-type hemorrhage was a strong predictor of poor prognosis in patients with large ischemic cores undergoing EVT. These results emphasize the importance of minimizing severe hemorrhagic transformation during reperfusion therapy.

Several limitations of this study should be acknowledged. First, this was a retrospective single-center analysis with a limited and relatively homogeneous sample. The sample sizes across several subgroups were too small to allow robust evaluation of the efficacy and safety of EVT. In particular, comparisons of sICH and mortality between groups were limited by the low number of events and inadequate statistical power. Therefore, these findings should be regarded as exploratory and require confirmation in adequately powered independent studies focused on EVT decision-making in patients with DWI-ASPECTS 3–5. Second, a large ischemic core was defined using the semi-quantitative DWI-ASPECTS score because of its practicality in emergency clinical settings. Although DWI-ASPECTS correlated with infarct volume (r_s_ = −0.502), the moderate strength of this correlation suggests that these two metrics are not interchangeable. Volumetric assessment may capture aspects of tissue injury not reflected by the semi-quantitative ASPECTS score, potentially offering superior prognostic discrimination. DWI-ASPECTS should not be used solely for clinical decision-making, but should be comprehensively evaluated in conjunction with clinical manifestations and multimodal imaging information. Future studies should incorporate automated DWI-based infarct volume measurement tools to reduce measurement variability and to identify potential infarct volume thresholds at which patients with a large ischemic core may benefit from EVT. Third, detailed vascular anatomical characteristics, such as vessel tortuosity, access route complexity, and collateral status, were not assessed. These factors may affect procedural success and clinical outcomes. Future prospective studies should systematically collect these anatomical variables to determine whether specific anatomical features modify the treatment effect of EVT in patients with large ischemic core infarction. Finally, patient selection in this study was based on MRI using DWI-ASPECTS. Although DWI provides better sensitivity for early infarct detection than NCCT, MRI is less widely available in many emergency settings and may require longer acquisition times, potentially delaying treatment. Furthermore, TOF-MRA has limited sensitivity for detecting distal vessel occlusions. These factors may limit the generalizability of our findings to centers where CT or CT perfusion imaging is the primary diagnostic modality. Future studies directly comparing outcomes in patients selected by DWI-ASPECTS versus CT perfusion-based criteria may help define the optimal imaging strategy for selecting patients with large ischemic core stroke for EVT.

## Conclusions

In this retrospective study, EVT was associated with improved 90-day functional outcomes in patients with large ischemic core infarction selected using DWI-ASPECTS, without a statistically significant increase in sICH or mortality compared with medical management alone. Intravenous thrombolysis bridging therapy was independently associated with favorable outcomes, whereas PH2-type hemorrhage was strongly associated with poor prognosis. These findings suggest that EVT may be beneficial in carefully selected patients with large ischemic core infarction identified by DWI-ASPECTS. However, larger prospective studies with longer follow-up are required to further evaluate the safety of EVT and to refine imaging-based patient selection strategies.

## Data Availability

The datasets analyzed during the current study are available from the corresponding author on reasonable request.

## Abbreviations

EVT: Endovascular thrombectomy
LVO: Large-vessel occlusion
ASPECTS: Alberta Stroke Program Early Computed Tomographic Score
RESCUE Japan LIMIT: Recovery by Endovascular Salvage for Cerebral Ultra-Acute Embolism–Japan Large Ischemic Core Trial
RCTs: Randomized controlled trials
TENSION: Endovascular Thrombectomy for Acute Ischemic Stroke With Established Large Infarct
ANGEL-ASPECT: Trial of Endovascular Therapy for Acute Ischemic Stroke With Large Infarct
SELECT2: Trial of Endovascular Thrombectomy for Large Ischemic Strokes
LASTE: Large Stroke Therapy Evaluation
DWI-ASPECTS: Diffusion-Weighed Imaging Alberta Stroke Program Early CT Score
NCCT: Non-Contrast Computed Tomography
ACALVOS: Acute anterior circulation large artery occlusive stroke
mRS: Modified Rankin scale
NIHSS: National Institutes of Health Stroke Scale
mTICI: Modified Thrombolysis in Cerebral Infarction
HIP AA: Health Insurance Portability and Accountability Act
BP: Blood pressure
ORT: Onset to reperfusion time
TOF-MRA: Magnetic resonance time-of-flight angiography
T2-FLAIR: T2 fluid-attenuated inversion recovery
ECASS: European Cooperative Acute Stroke Study
HI: Hemorrhagic infarction
PH: Parenchymal hematoma
rt-PA: Recombinant tissue-type plasminogen activator
MT: Mechanical thrombectomy
CTP: Computed Tomography Perfusion
